# Clinical Applications of Patient-Specific 3D Printed Models in Cardiovascular Disease: Current Status and Future Directions

**DOI:** 10.3390/biom10111577

**Published:** 2020-11-20

**Authors:** Zhonghua Sun

**Affiliations:** Discipline of Medical Radiation Sciences, School of Molecular and Life Sciences Curtin University, GPO Box, U1987, Perth 6845, Australia; z.sun@curtin.edu.au; Tel.: +61-8-9266-7509; Fax: +61-8-9266-2377

**Keywords:** 3D printing, computed tomography, image processing, medicine, model, segmentation, visualization

## Abstract

Three-dimensional (3D) printing has been increasingly used in medicine with applications in many different fields ranging from orthopaedics and tumours to cardiovascular disease. Realistic 3D models can be printed with different materials to replicate anatomical structures and pathologies with high accuracy. 3D printed models generated from medical imaging data acquired with computed tomography, magnetic resonance imaging or ultrasound augment the understanding of complex anatomy and pathology, assist preoperative planning and simulate surgical or interventional procedures to achieve precision medicine for improvement of treatment outcomes, train young or junior doctors to gain their confidence in patient management and provide medical education to medical students or healthcare professionals as an effective training tool. This article provides an overview of patient-specific 3D printed models with a focus on the applications in cardiovascular disease including: 3D printed models in congenital heart disease, coronary artery disease, pulmonary embolism, aortic aneurysm and aortic dissection, and aortic valvular disease. Clinical value of the patient-specific 3D printed models in these areas is presented based on the current literature, while limitations and future research in 3D printing including bioprinting of cardiovascular disease are highlighted.

## 1. Introduction

Although three-dimensional (3D) printing is not a new technology, it has experienced rapid developments over the last decade with increasing studies available in the literature documenting its clinical value in different areas. Patient-specific or personalized 3D printed models accurately replicate normal anatomical structures and pathologies, thus serving as a valuable tool in medical applications ranging from the initial orthopaedics and maxillofacial domain to cardiovascular disease and tumours [1,2,3,4,5,6,7,8,9,10]. 3D printed physical models provide useful information in pre-operative planning and simulation of complex surgical procedures, education of medical students and residents, intraoperative orientation, and physician-patient communication [8,9,10,11,12,13,14].

Evidence of 3D printed models in these applications is dominated by case reports and case series, while multi-centre studies and randomized controlled trials (RCTs) are increasingly reported in the literature [15,16,17,18,19,20]. 3D printed models augment clinicians’ confidence in managing different clinical situations by improving their understanding of complex spatial relationship between the normal anatomy and pathologies which cannot be accurately achieved by the traditional image visualizations. With growing interest of 3D printing in the medical community, guidelines are beginning to emerge about the appropriate use of 3D printing for medical scenarios as recommended by several societies such as the Radiological Society of North America (RSNA) 3D printing Special Interest Group or the new Society for Cardiovascular Magnetic Resonance (SCMR) 3D working group [21,22]. Thus, it is important to have a clear understanding of the clinical value of 3D printing and potential limitations.

**Figure 1:**
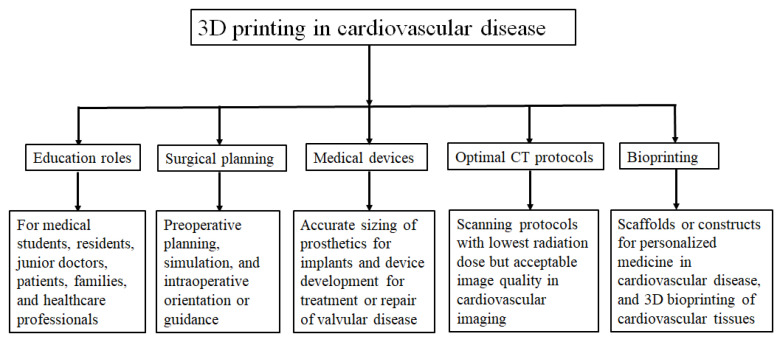
Flow chart shows 3D printing applications in medicine covering a range of areas.

The review article aims to provide an overview of 3D printed models in medical applications, specifically focusing its value in cardiovascular disease since its clinical applications in orthopaedics and maxillofacial areas are well explored and confirmed in the literature [23,24,25]. Further to the commonly reported usefulness of 3D printed models, use of 3D printed models for developing optimal computed tomography (CT) scanning parameters in cardiovascular disease is discussed and future research directions are highlighted. Figure 1 is a summary of 3D printing in the cardiovascular applications.

## 2. Steps to Create 3D Segmentation Volume Data for 3D Printing

**Figure 2:**
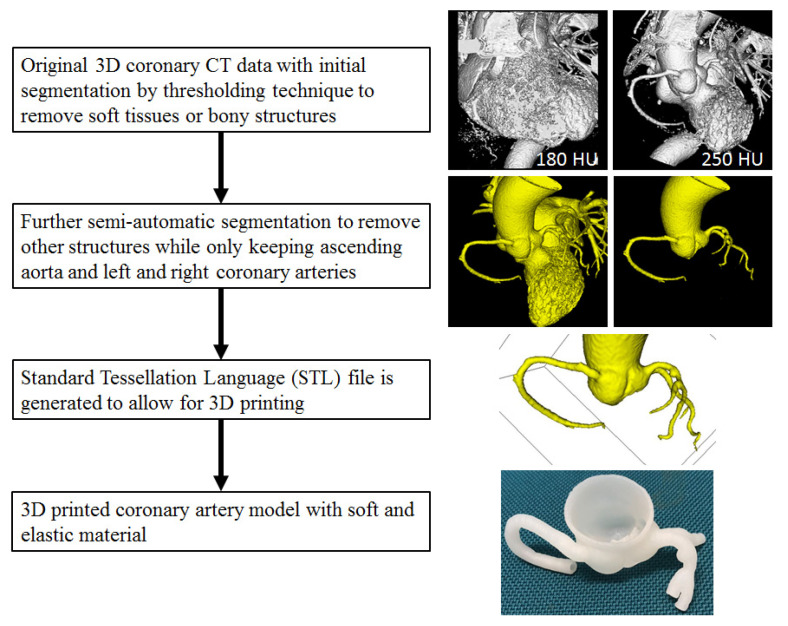
Steps to create 3D printed model from segmentation of original coronary CT volume data to generation of segmented data containing only regions of interest (coronary artery tree and ascending aorta in this example) and STL file for 3D printing. The coronary model was printed with Visijet M2 ENT material by 3D Systems.

The first and important step to generate a 3D printed model is to acquire high-quality imaging data, mainly CT or magnetic resonance imaging (MRI) volumetric images, although ultrasound or invasive angiography images are also used in some studies for 3D printing [11,12,13]. Volume datasets first undergo a series of image post-processing and segmentation (automatic or semi-automatic segmentation) steps to remove unwanted or irrelevant structures while keeping the regions of interest for 3D printing. Semi-automatic or manual editing is usually needed for further editing to only preserve the anatomical structures that are finally used for 3D printing. Figure 2 is a flow chart showing the steps from original imaging data editing or segmentation to create a Standard Tessellation Language (STL) file and print a physical model.

The physical model can be printed with different materials, depending on many factors, such as clinical purpose and requirements, availability of 3D printers and printing materials and associated costs. Readers are referred to some review articles providing a good summary of the 3D printing technologies and 3D printed models with use of a range of printing materials [11,12,13,14,26]. Some of the materials used for printing cardiovascular tissues in terms of mechanical properties and biocompatibility will be discussed in the section of bioprinting.

## 3. Clinical Applications of 3D Printing in Cardiovascular Disease

3D printing has shown great promise in many medical applications covering a spectrum of areas, depending on the location, type and severity of pathologies. For example, in the domain of tumour diagnosis and treatment, its application mainly lies on the pre-surgical planning and simulation or intraoperative orientation to guide surgical procedures, thus achieving more effective outcomes while reducing complications [14,27,28]. Whereas in the field of cardiovascular disease, in particular, congenital heart disease, the clinical value of 3D printing focuses on education of medical students or junior doctors or residents, improves physician-patient communication and increases confidence of cardiologists or cardiac surgeons in dealing with complex cardiac conditions [10,11,12,13,26]. 3D printing is useful to develop medical devices in the treatment of aortic or valvular diseases [26,29,30,31,32]. In the following sections, clinical value of 3D printing is discussed with regard to each specific area based on the current literature.

### 3.1. 3D Printing in Congenital Heart Disease

Since 3D printing can provide an outstanding view of complex anatomical details, it has played an important role in treating patients with congenital heart disease (CHD). Diagnosis and treatment of CHD requires a comprehensive and good understanding of complex spatial relationship between normal cardiac anatomy and disease, and 3D printing is a transformative technology that has shown to change the current practice in the management of CHD.

**Figure 3:**
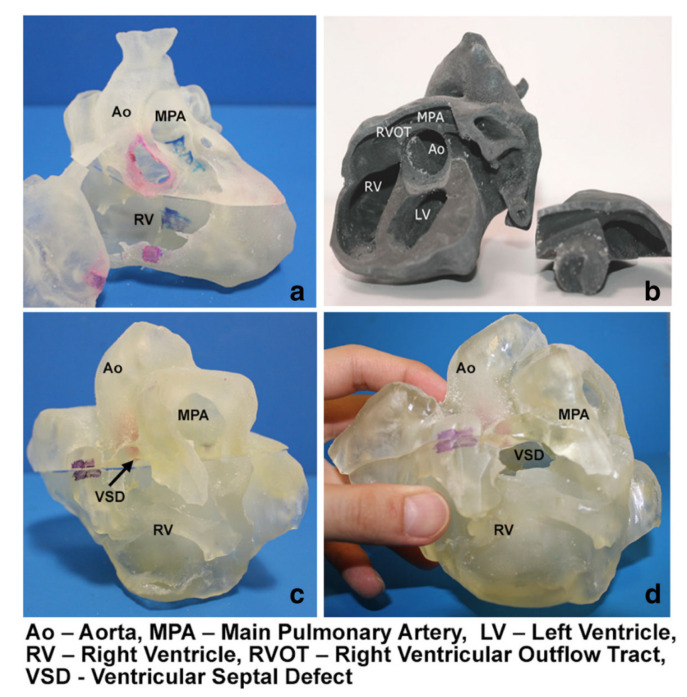
3D printed heart models showing normal anatomy and pathology. (**a**) Normal heart model created from cardiac CT and is partitioned into three pieces allowing visualization of interventricular septum. (**b**) Repaired Tetralogy of Fallot (ToF) from an adult patient. The model was created from cardiac magnetic resonance imaging (MRI) and separated into two pieces allowing for clear visualization of overriding aorta and pulmonary infundibular stenosis. (**c**) Unrepaired ToF heart model from an infant. The model was created from 3D echocardiographic images and partitioned into two pieces showing the ventricular septal defect (VSD). (**d**) Unrepaired ToF heart model from an infant with superior and inferior portions showing VSD and the aortic overriding in relation to the VSD. Reprinted with permission under open access from Loke et al. [17].

Precise pre-surgical planning of CHD can be achieved with use of 3D printed models, and this is reported in recently published systematic reviews and meta-analyses and a number of other studies [13,33,34,35,36,37,38,39,40]. Of 24 eligible studies in the systematic review conducted by Lau and Sun, 15 of them reported the 3D printed models in the pre-operative planning of CHD treatment, provided how 3D printed models assisted the planning of surgical procedures and surgeons’ opinion on the 3D printed models [13]. The 3D printed models were found to assist surgeons define the best surgical approach, in particular in dealing with complex CHD cases such as double outlet right ventricle (DORV) (Figure 3) [13]. Personalized therapeutic strategy can be achieved and the best surgery option can be made with the aid of patient-specific 3D printed models as reported by a recent study [41].

**Figure 4:**
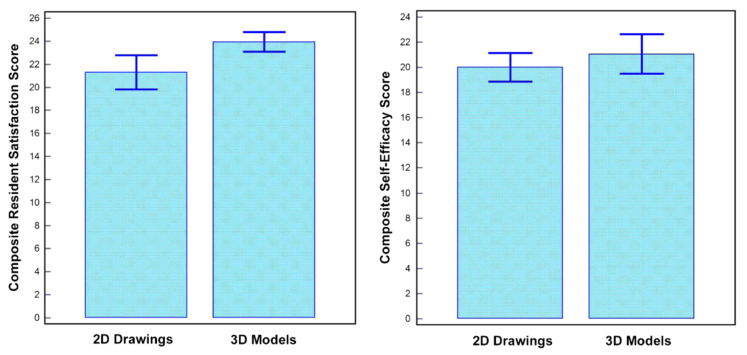
Impact of 3D printed heart models on medical education. Improvement was found in residents’ knowledge on congenital heart disease with use of 3D printed models when compared to 2D images. A statistically significant difference was noticed in satisfaction ratings in the group having 3D printed heart models when compared to the control group (*p* = 0.03). While residents in the 3D printed model group had higher self-efficacy scores (self-reported confidence), this did not reach significant difference compared to the control group using 2D images/drawings (*p* = 0.39). Reprinted with permission under open access from Loke et al. [17].

Another common application of 3D printing in CHD is about its usefulness in medical education. This is noticed in 50% of the studies in the same systematic review [13]. Of these studies, there are four RCTs comparing the 3D printed models with traditional teaching methods in CHD for medical students and residents [16,17,18,42]. While the study by Wang et al. did not show any significant improvement in the 3D printing group when compared to the control group [42], the other three studies indicated that 3D printed models served as a useful tool in teaching and learning of complex CHD situations as opposed to the control group using standard teaching tools (Figure 4) [16,17,18]. This is consistent with other studies reporting the utility of 3D printed models in the education of healthcare professionals, patients or families [43,44,45].

Less commonly reported areas but also showing clinical value of 3D printing in CHD include communication between doctor and patients or parents of patients, and pre-surgical simulation. Despite only a few studies available with qualitative results, most of the participants agreed to the conclusion that 3D printed models augmented doctor-patient or doctor-doctor communication. High satisfaction was found with use of 3D printed models during patient’s consultation with their doctors [17,36,43]. This highlights the complementary role of 3D printed models in improving the doctor-patient communication.

Pre-surgical simulation is the ultimate goal of surgical planning as it guides performing the complex surgical procedures, thus leading to improved outcomes in the patient treatment. This is especially useful for inexperienced surgeons or junior doctors to practice surgical simulation procedures on 3D printed models, thus creating intracardiac pathways [46].

### 3.2. 3D Printing in Structural Heart Disease and Cardiac Interventions

3D printed models have potential value in structural heart disease, especially in interventional cardiology through simulation of cardiac interventions to determine feasibility of interventional procedures, selection of appropriate device, predict procedure-related outcomes and follow-up of post-procedural device deployment [34,35,47,48,49]. Specifically, 3D printed models have played an important role in the planning of treatment of valvular disease and left atrial appendage closure [47,48,49]. A systematic review of 29 articles on 3D printing in heart valve disease shows that the most common application of 3D printing is the preoperative planning of valvular disease (63%), followed by surgical training (19%) and device testing and development (11%) [50].

Wang and colleagues in their recent systematic review analysed 43 studies about the applications of 3D printed models in adult cardiovascular disease treated with surgeries or catheter-based interventional procedures [51]. Of 43 studies, most of them (35/43, 81%) focused on the clinical applications of 3D printed models in adult cardiovascular disease, primarily assisting cardiovascular surgery and transcatheter interventions. Of different clinical scenarios, 3D printing was reported as a useful tool in preoperative planning and simulation of the two common heart diseases, left atrial appendage occlusion (LAAO) and transcatheter aortic valve replacement (TAVR). Of 296 patients included in these 35 studies, half of them and 17.6% underwent the LAAO and TAVR, respectively [51]. For the remaining eight students, 3D printed models were used for simulation and training of aortic valvular diseases to improve surgical trainees’ understanding of the disease.

Another application of 3D printing lies in optimal preoperative TAVR planning confirmed by a recent study reporting the potential value of using 3D printed models for assessment of paravalvular leak (PVL) [52]. Thorburn et al. created five 3D printed models consisting of aortic root, coronary ostium and left ventricular outflow tract based on cardiac CT images. The printed models were connected to a closed pressure system for measuring PVL following TAVR implantation. The amount of PVL measured in 3D printed models was compared to that detected on echocardiography in these patients who were treated by TAVR. Their analysis showed a significant correlation of the paravalvular leak volume between 3D printed models and postoperative echocardiographic measurements in patients [52]. This study highlights the potential value of utilizing 3D printed models in predicting the paravalvular leak in patients treated with TAVR, although future studies are needed to look at more areas, such as testing different valve sizes and implantation of the devices in different positions.

**Figure 5:**
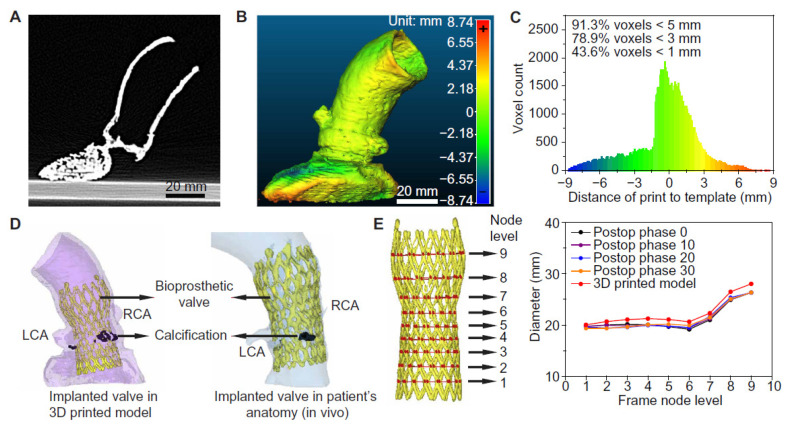
Anatomical fidelity analyses of the 3D printed aortic root models and comparison to patient postoperative data. (**A**) CT scan of the 3D printed aortic root model. (**B**) Calibrated distance map comparing the anatomical fidelity of the 3D printed aortic root model with the patient’s anatomy. (**C**) Histogram of the calibrated distances between the surface points of the 3D printed aortic root model and the patient’s anatomy. (**D**) Comparison of the implanted TAVR prosthesis in the 3D printed model with the patient’s postoperative data. RCA-right coronary artery; LCA- left coronary artery. (**E**) Comparison of changes in frame diameters of the implanted valve in the 3D printed model with the patient’s postoperative data at nine different node levels. Reprinted with permission under open access from Haghiashtiani et al. [53].

**Figure 6:**
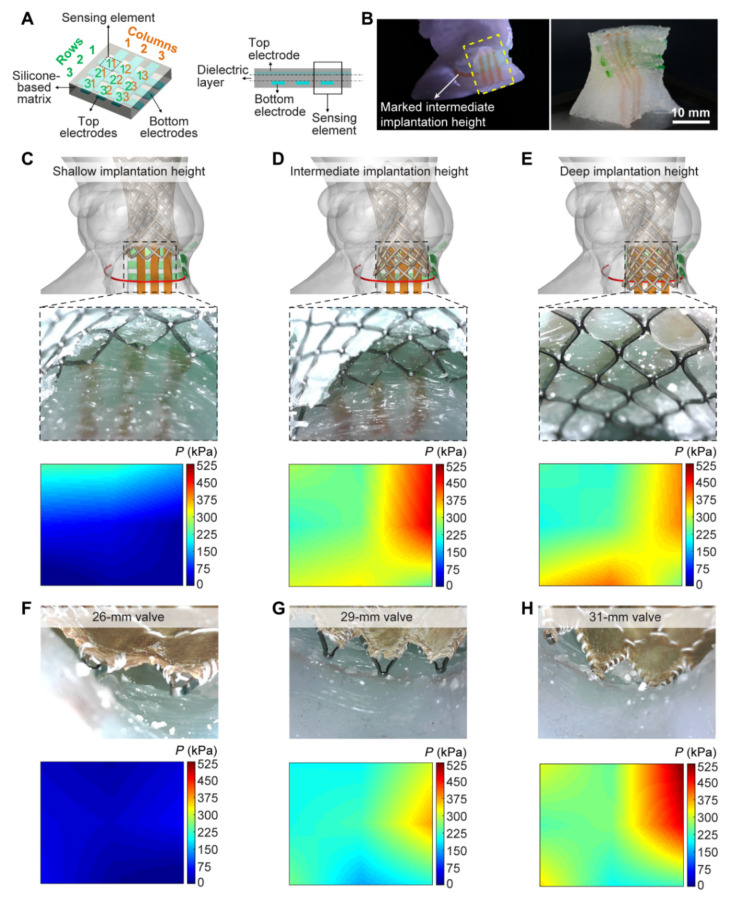
3D printed aortic root model with internal sensor arrays and visualization of applied pressures after valve implantation. (**A**) Schematic of the sensor array concept design in planar configuration. (**B**) 3D printed aortic root model with internal sensor array (left) and the corresponding isolated sensor region (right). The vertical (orange) and horizontal (green) electrodes of the integrated sensor arrays on the model correspond to the top and bottom electrodes in the planar design, respectively. (**C**) Implantation of the 29-mm Evolut R TAVR valve frame at a shallow height. (**D**) Implantation of the 29-mm Evolut R TAVR valve frame at an intermediate height. (**E**) Implantation of the 29-mm Evolut R TAVR valve frame at a deep height. The red marked lines in (**C**–**E**) correspond to the intermediate implantation height. (**F**) Implantation of the 26-mm Evolut R TAVR valve at an intermediate height. (**G**) Implantation of the 29-mm Evolut R TAVR valve at an intermediate height. (**H**) Implantation of the 31-mm CoreValve TAVR valve at an intermediate height. Reprinted with permission under open access from Haghiashtiani et al. [53].

Haghiashtiani et al. further extended the 3D printed aortic model to an advanced level of integrating internal sensors array within the aortic root, thus showing the feasibility of creating dynamic functionalities of the 3D printed models [53]. Authors created two patient-specific aortic root models using CT images with each model consisting of anatomical structures, such as the aortic wall, myocardium, aortic valve leaflets and calcifications. These two models served two purposes with model 1 used for experiments of fidelity analysis (Figure 5), while model 2 was used for conducting hemodynamic analysis following TAVR (Figure 6). Four inks were used to print different anatomical structures representing different properties, including the ink for aortic wall, myocardium and leaflets and calcified region as well as for the supporting material. For production of the models with integrated sensors in the aortic root, finer nozzles and layer height of 0.4 mm with high resolution were used to print the sensor array in the lower section of the model (Figure 6). A bioprosthetic valve was deployed into the 3D printed models to simulate TAVR treatment. Results showed the accuracy of 3D printed model replicating patient’s anatomy and physical behaviour as demonstrated by the postoperative data at different cardiac phases (Figure 6D,E). Hemodynamic analysis validated the potential value of 3D printed models with internal sensors for prediction of conduction disturbances by providing quantitative assessments of pressure changes in relation to different valve sizes (Figure 6F–H). Findings of this study confirm the promising role of 3D printed realistic models for minimizing the risks associated with TARV procedures, thus assisting the development of optimal medical devices.

### 3.3. 3D Printing in Coronary Artery Disease

Use of 3D printed models in coronary artery disease (CAD) is mainly shown to guide treatment of complex coronary anomalies and this is dominated by case reports in the literature [54,55,56,57]. These isolated case reports showed the usefulness of 3D printed coronary models to simulate interventional coronary procedures, plan and guide treatment strategy of complex coronary disease. Another application of 3D printed coronary artery models is to improve understanding of coronary anomalies. Lee et al. in this study selected eight cases with one normal coronary artery and seven diseased coronary arteries comprising different coronary abnormalities [58]. Eight patient-specific coronary models were created and presented to nine cardiovascular researchers and eight clinicians (two cardiac surgeons and six cardiologists) with the aim of seeking their feedback on the usefulness of 3D printed models. Both groups indicated that 3D printed models enhanced understanding of coronary anatomy and pathologies, while the clinical group with more imaging experience indicated that 3D printed models are more useful than CT images alone. Further, this study suggested that 3D printed coronary models are useful in other areas such as improving patient-physician communication by better explaining conditions to patients; preoperative planning for cardiac surgeons and improving decision-making by multidisciplinary team collaboration through demonstrating the diagnostic anatomy to clinicians.

**Figure 7:**
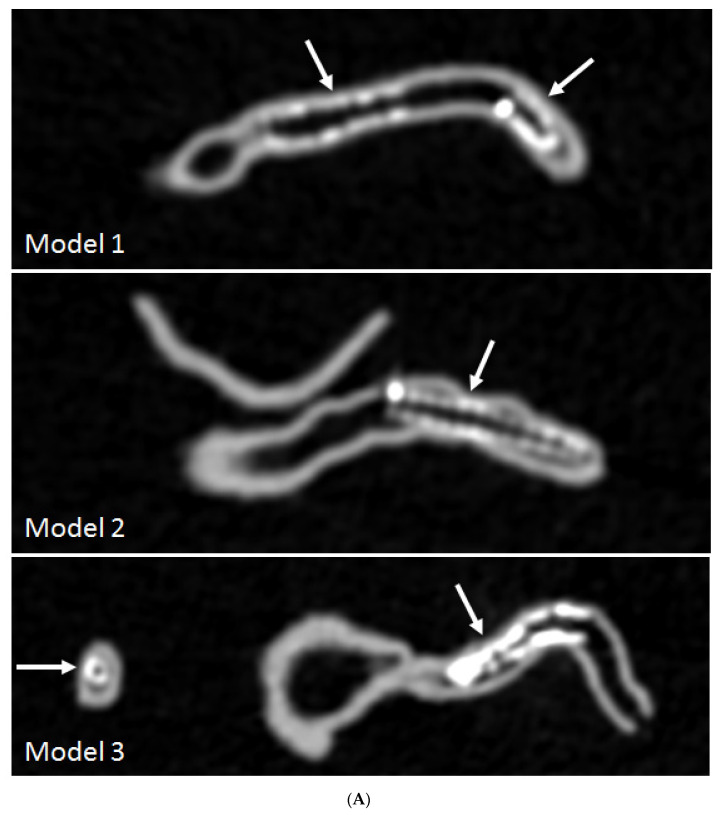

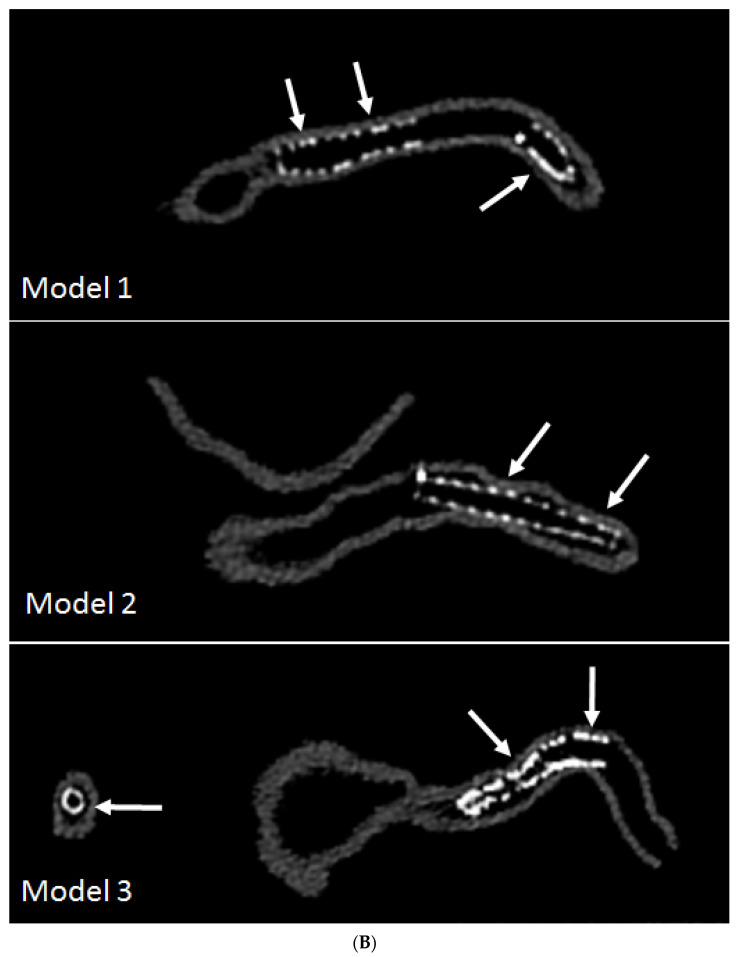
3D printed coronary models with coronary stenting scanned on 192-slice CT scanner and images reconstructed with soft and sharp kernel algorithms. (**A**) images were reconstructed with standard soft kernel Bv36 algorithm showing that right coronary stents in these 3 models and left anterior descending stent (in model 3). (**B**) images were reconstructed with sharp kernel Bv59 of the same models with significant improvement of visualization of the stents and stented lumen, in particular improved visualization of stent wires in models 2 and 3. Arrows refer to the stent wires. Reprinted with permission under open access from Sun and Jansen [63].]

**Figure 8:**
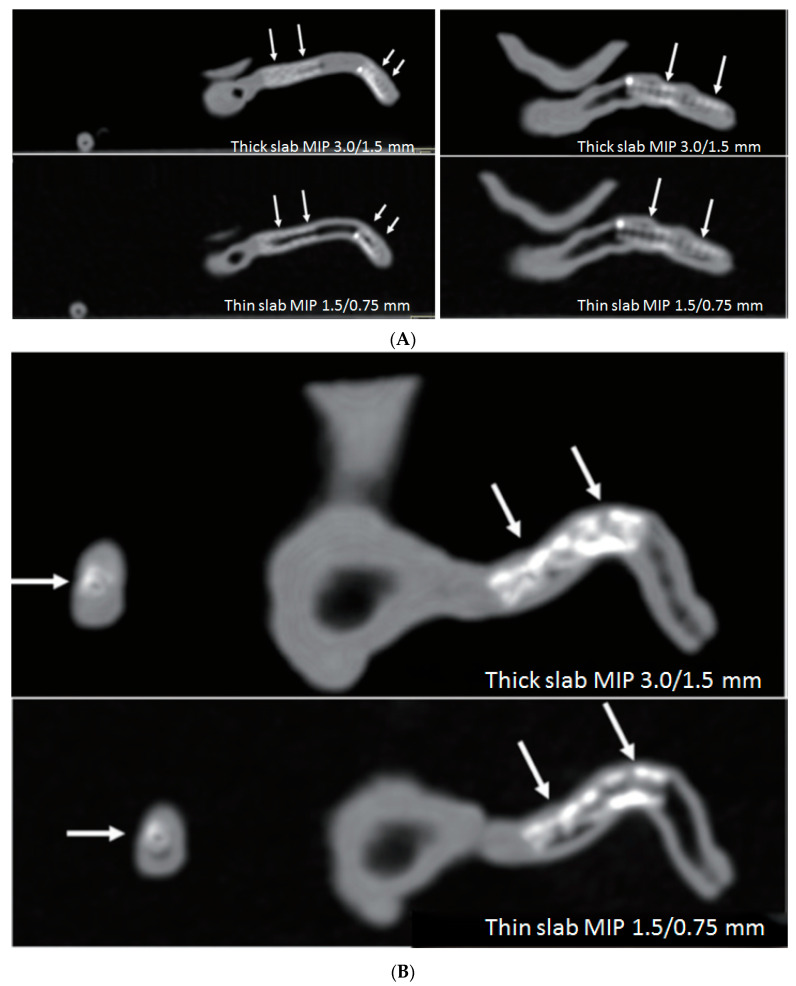
Comparison of thick slab with thin slab maximum-intensity projection (MIP) images for visualization of stents and stented lumen in 3D printed models of right coronary stents. (**A**) Thin slab MIP images improve assessment of the stented lumen compared to thick slab MIP images (arrows), and this is apparent in the model 3 (**B**) in the presence of multiple stent wires (arrows). Reprinted with permission under open access from Sun and Jansen [63].

**Figure 9:**
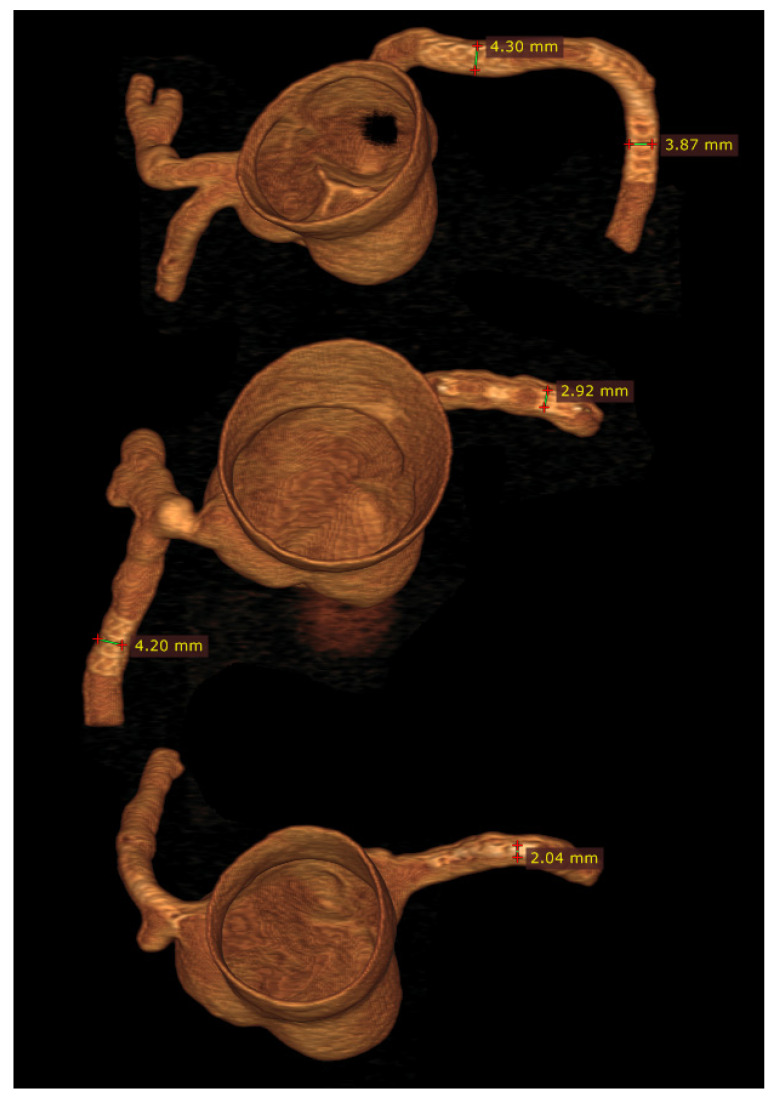
3D volume rendering images of 3D printed coronary models with stents placed in the coronary arteries. Reprinted with permission under open access from Sun and Jansen [63].

An emerging research area of using 3D printed coronary models is to investigate optimal coronary CT scanning protocols for visualization of calcified plaques and coronary lumen in coronary stenting. Some recent studies by Sun and colleagues reported their experience with promising results achieved through use of personalized coronary models [59,60,61,62,63]. High-resolution CT imaging allows for better visualization and assessment of coronary lumen stenosis caused by calcified plaques, thus further validating previous findings about the effect of spatial resolution on diagnostic assessment of calcified coronary plaques [60]. Coronary CT angiography is widely used in the diagnosis of CAD, however, its clinical value in coronary stenting is debatable due to the beam hardening artifacts arising from coronary stents which negatively affect the accurate assessment of coronary lumen, especially the assessment of in-stent restenosis. 3D printed coronary artery models with six different stent diameters placed in the coronary arteries were used to simulate coronary stenting in the study conducted by Sun and Jansen [63]. With scans conducted on a latest 192-slice CT scanner, results showed that images reconstructed with a sharp kernel algorithm significantly improved the stent and stented lumen visibility compared to images reconstructed with a standard kernel algorithm (Figure 7). Thin slab maximum-intensity projection (MIP) images allowed for better visualization of stented lumen in comparison with thick slab MIP images (Figure 8). 3D volume rendering images provide clear views of the stent location in relation to the coronary anatomy as shown in Figure 9. These preliminary reports along with others suggest the potential value of using 3D printed coronary or cardiac models for studying the optimal CT protocols, although further research is needed to validate these findings [59,62,63].

**Figure 10:**
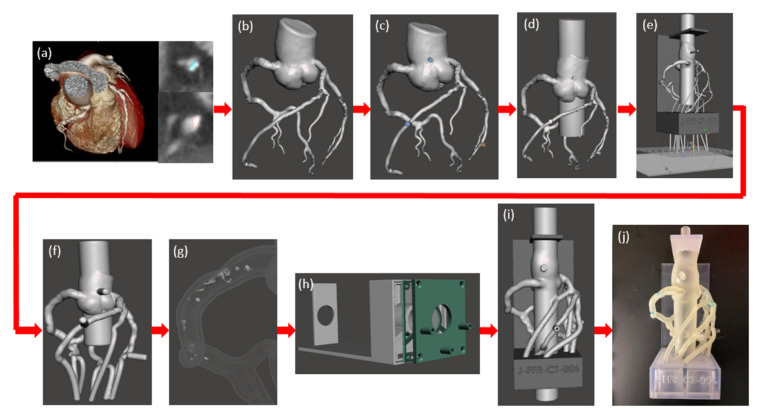
Model development process of 3D printed coronary artery model and experimental setup. (**a**) Coronary CT angiography scans of the heart tissue and the three main coronary arteries were imported for analysis. The coronary arteries were segmented separately from the calcification using thresholding and contouring methods. (**b**) A stereolithographic file was exported from volume data and imported into Autodesk Meshmixer and segmentation errors were removed. (**c**) Cylindrical meshes were appended to the aortic root and the diseased coronary artery for future pressure sensor connections. (**d**) The aortic root was extended at both the inlet and the outlet. (**e**) Vessel branches were extended through each of the three chambers and a plane cut was administered at the vessel outlets for parallel ends. (**f**) A 2mm wall was generated and the lumen was hollowed out. (**g**) The calcification was solidified and subtracted from the vasculature. (**h**) A three-chamber support structure was imported into Autodesk Meshmixer. (**i**) Then model and support structure are aligned and ready to be printed. (**j**) The model is 3D printed, cleaned, and ready to be attached to a flow loop. Reprinted with permission under open access from Sommer et al. [64].

**Figure 11:**
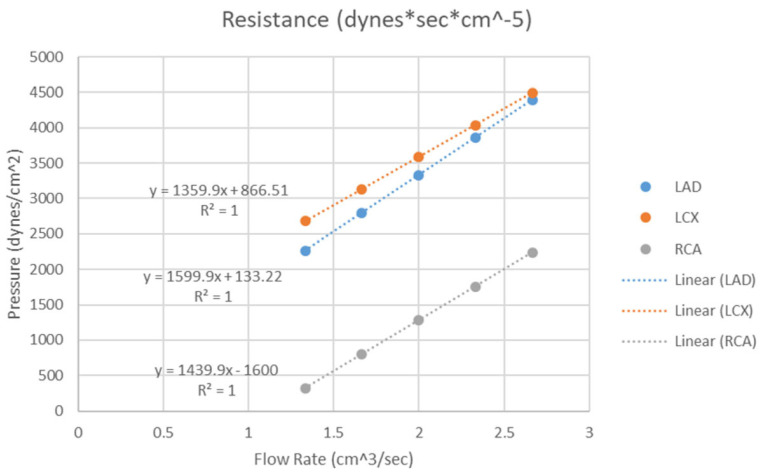
Three chamber resistance. The resistance of the chamber was determined by recording the pressure through a tube at flow rates from 1.33–2.66 cm^3^/sec within the LAD, LCX and RCA chambers. LAD-left anterior descending, LCX-left circumflex, RCA-right coronary artery. Reprinted with permission under open access from Sommer et al. [64].

Further to the above-mentioned applications, 3D printed coronary artery models can also be used to simulate blood flow to the coronary arteries with regard to the hemodynamic changes associated with the development of atherosclerosis. Sommer and colleagues created 3D printed coronary models aiming to simulate the distal resistance and compliance of the coronary arteries [64]. Figure 10 is a workflow showing the steps from creation of the 3D segmented coronary artery tree to 3D printed model for investigation of the hemodynamic flow. The vasculature of the model was printed with soft and elastic material, Agilus, reflecting the arterial compliance, while the calcification and other supporting structure were printed with hard and rigid material, Vero. A 3D printed chamber was designed to enable the 3D printed coronary model connected to a physiological flow pump generating a constant flow loop. A range of catheters with different diameters and lengths were connected to the support chamber allowing for simulation of hemodymics in the thoracic aorta and coronary arteries at rest, light and moderate exercise situations. Five personalized 3D printed coronary models were created in their study with successful measurements of pressure at flow rates ranging from 80 to 160 mL/min at three main coronary arteries, including right coronary artery, left anterior descending and left circumflex arteries. Results showed the resistance of the chamber for these three main coronary arteries was negligible, with mean resistance being 0.65–5.86%, 1.23–6.86% and 0.05–1.67%, respectively corresponding to these coronary arteries (Figure 11). This study developed an innovative method to simulate realistic physiological blood flow in the coronary arteries using 3D printed models, thus creating benchtop models for further comprehensive analysis and simulation of coronary flow changes.

### 3.4. 3D Printing in Aortic Aneurysm and Aortic Dissection

Clinical value of 3D printing in aortic disease, mainly aortic aneurysm and dissection is also dominated by isolated case reports showing its application of creating realistic aortic models for simulation of surgical procedures, in particular minimally invasive endovascular stent grafting procedures of treating abdominal aortic aneurysm (AAA) and aortic dissection [65,66,67,68,69,70,71,72,73,74]. Tam et al. analysed 42 studies in their systematic review, with nearly 50% of them (20 studies) related to 3D printing in AAA, eight out of 42 studies on thoracic aorta pathology, and the remaining reports on 3D printing in other vascular diseases, such as carotid, subclavian, celiac, and femoral arteries [75].

**Figure 12:**
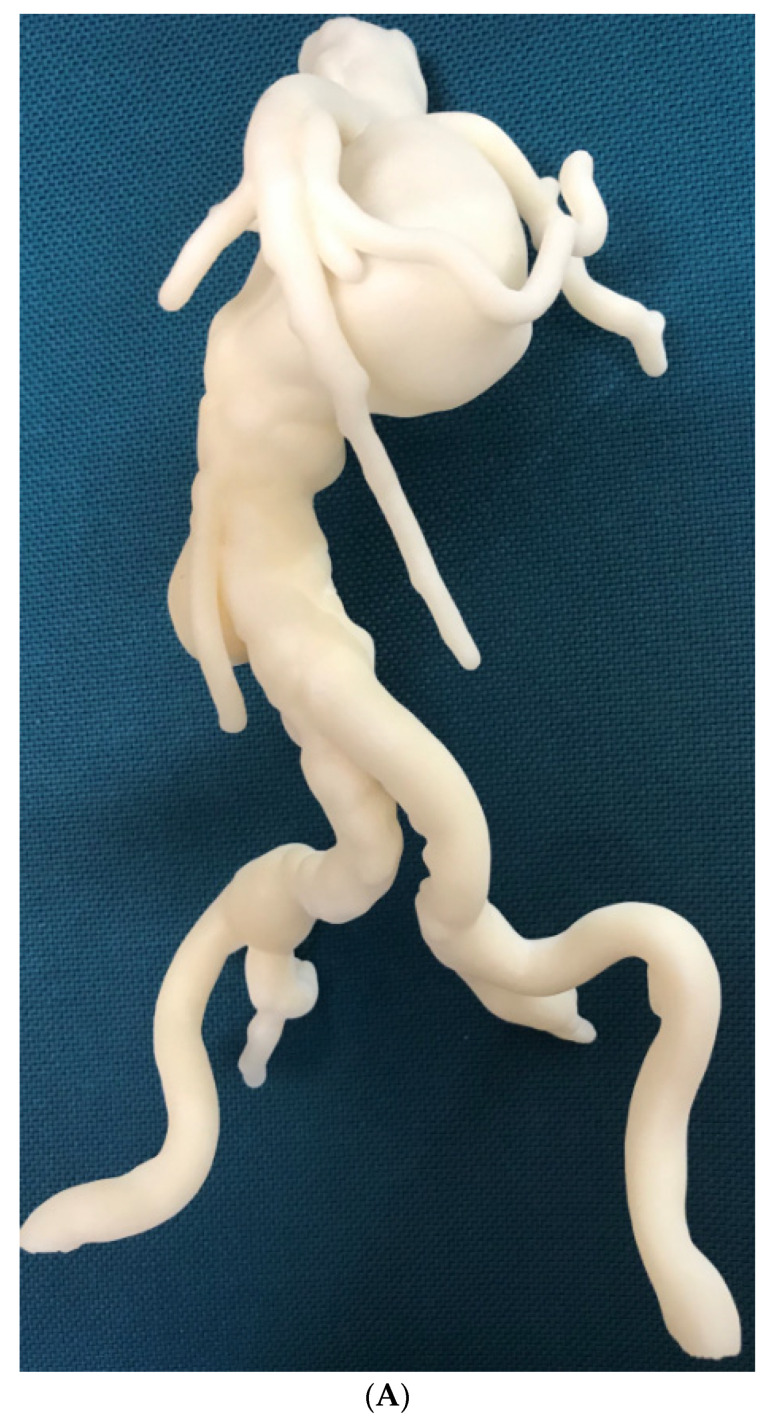

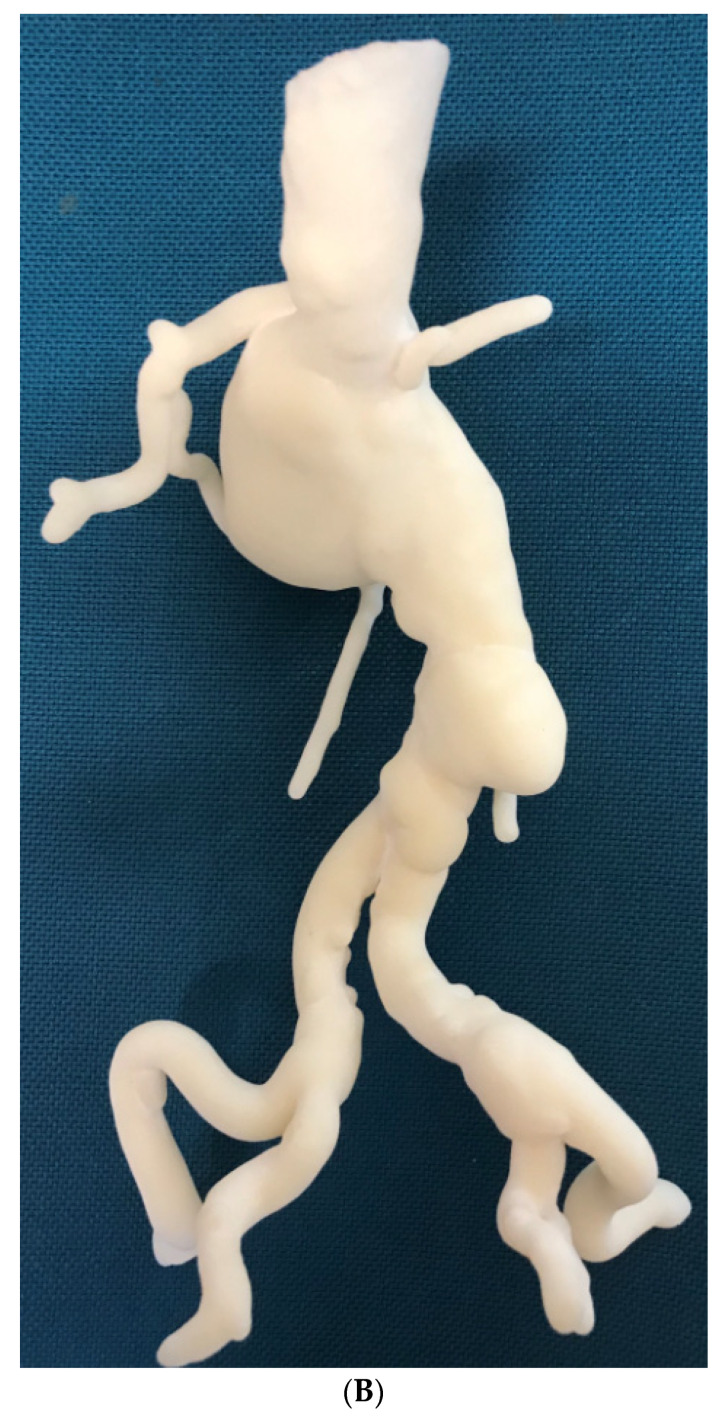
3D printed model of abdominal aortic aneurysm from CT angiographic data. (**A**) Anterior review of the 3D printed AAA model showing an infrarenal AAA extending to common iliac arteries. (**B**) Posterior view of the aneurysm and renal arteries. The mode was printed with ‘Strong and Flexible Plastic’ material by Shapeways.

Patient-specific 3D printed aortic models accurately replicate aortic aneurysm and aortic dissection based on patient’s CT imaging data, although some measurement differences at the aortic locations and true lumen are reported to be more than 1.0 mm (Figure 12) [65,66]. Most of the current applications of 3D printed aorta models are primarily related to the preoperative planning and simulation of endovascular repair of AAA, especially in dealing with complex cases, such as fenestrated stent grafts or aortic arch aneurysm [68,69,70,71,72,73]. These situations present challenges for vascular surgeons to manage the patients, thus 3D printed models serve as an additional tool to simulate endovascular aortic repair procedures and facilitate decision-making. Huang et al. first reported the use of 3D printed model to guide stent grafting fenestrations in a juxtarenal aortic aneurysm [69]. A 3D printed skin template based on CT data was created to locate the fenestration position on the stent graft. The skin template was then used to cover the stent graft prior to the surgical procedure for providing accurate location of fenestration holes on the stent graft. This novel approach for improving fenestration accuracy on the stent graft was further confirmed by Rynio et al. who produced a 3D printed aortic arch template from CT angiographic data to improve accuracy and effective management of thoracic aortic aneurysms by the fenestrated and modified stent grafts [74].

Karkkainen and colleagues created a realistic 3D printed AAA model for simulation of endovascular aneurysm repair by surgical trainees with different experiences [76]. Their 3D printed AAA model consists of three layers: inner rigid layer and soft flexible outer layer with each having 3 mm thickness and the thin 1 mm inside layer covering the rigid one. The model was connected to a fluid pump with endovascular stent grafting procedures performed under angiography. Twenty trainees (13 were less experienced and seven were experienced) participated in performing 22 simulations with experienced trainees having significantly lower procedural time and fluoroscopic exposure time than inexperienced ones (*p* < 0.05). All experienced trainees completed the procedures independently and in less than 45 min, in contrast, only two of the less experienced trainees completed the entire procedures independently and six out of 13 completed the procedures in less than 45 min. This study presents another opportunity of using 3D printed aorta models for simulation and training of surgical procedures [76].

**Figure 13:**
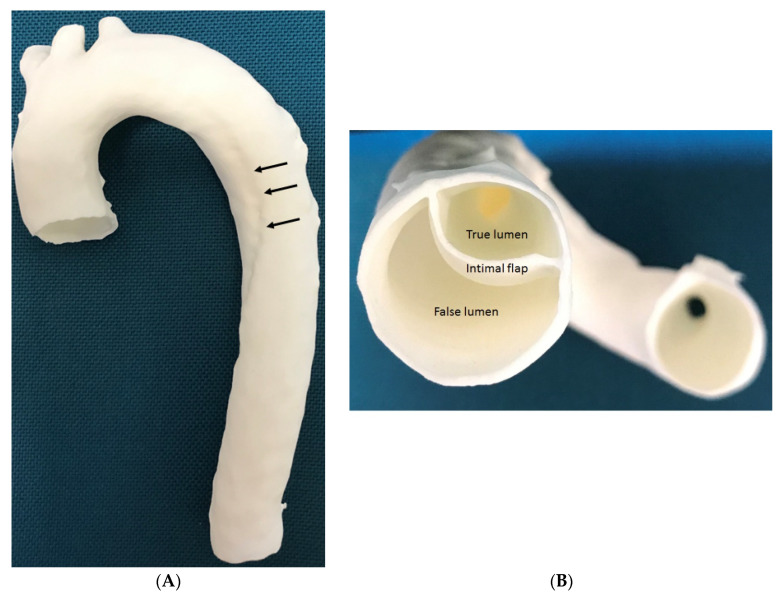
3D printed model of type B aortic dissection. (**A**) Anterior view of the 3D printed model of aortic arch and descending aorta. Arrows refer to the intimal flap separating true lumen from false lumen. (**B**) Inferior view of the intimal flap and true and false lumen compartments. The mode was printed with ‘strong and flexible plastic’ material by Shapeways.

3D printing models of aortic dissection, in particular, reproducing the intimal flap is very challenging due to the very thin structure separating true lumen from false lumen (Figure 13) [64,65]. Limited research is done in this area and a study by Hossien et al. reported interesting findings [67]. Three cases of type A dissection were selected with involvement of aortic branches to some extent. 3D aortic models were printed using polylactic acid materials demonstrating true and false lumen in relation to the aortic branches. Intimal flap was also printed to separate true lumen from the false lumen in their case report. Similar to other aortic disease, the 3D printed models allow for surgical simulation of complex endovascular repair of aortic dissection, although further research is needed to test its clinical value in more cases.

**Figure 14:**
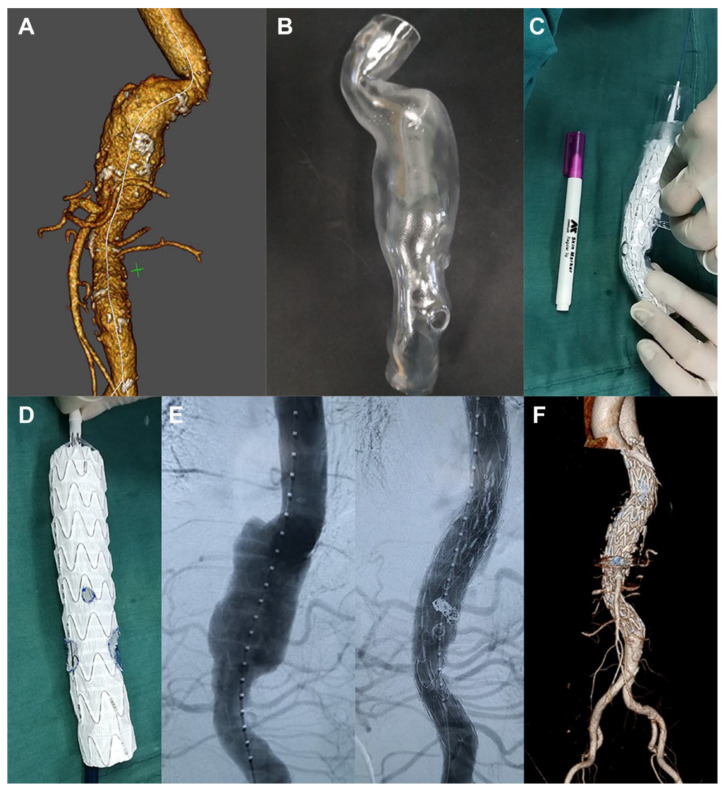
Use of 3D printed model to guide fenestrated stent graft repair of a thoracoabdominal aortic aneurysm (TAAA). (**A**) According to the measurements determined in EndoSize or 3mensio, (**B**) a 3D model of the TAAA was printed. (**C**) The main aortic stent-graft was completely released in the 3D model, and the position of each fenestration or branch was marked. (**D**) The fenestration was cut using an electric pen, and a wire was sutured around its edge. (**E**) Intraoperative images showing the calibrated catheter (left) in the TAAA and the fenestrated stent-graft (right) in position. (**F**) Completion of the procedure with addition of the proximal stent-graft. Reprinted with permission from Tong et al. [77].

**Figure 15:**
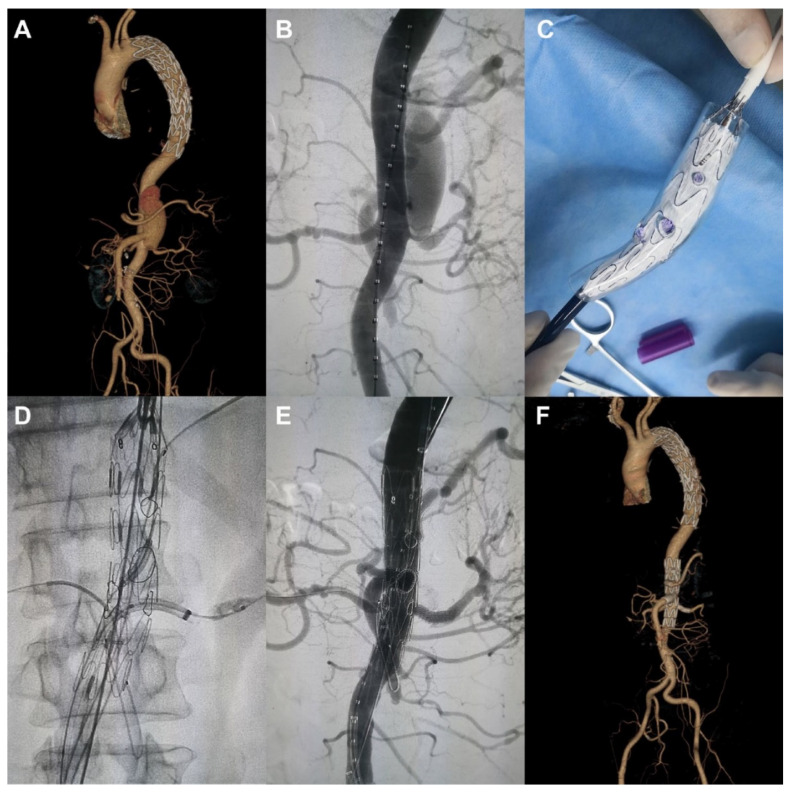
Use of 3D printed model to guide fenestrated stent graft repair of a thoracoabdominal aortic dissection. (**A**,**B**) A thoracoabdominal aortic dissection after prior thoracic endovascular aortic repair. (**C**) The main aortic stent-graft was completely released in the 3D model of the lesion, and the position of each fenestration or branch was marked. (**D**,**E**) Intraoperative images showing the fenestrations for the visceral branches. (**F**) Completed procedure showing the bridging stentgrafts in the target vessels and obliteration of the false lumen. Reprinted with permission from Tong et al. [77].

Tong et al. expanded the application of 3D printing aorta models to guide surgical planning of fenestration for treatment of patients with thoracoabdominal aortic aneurysm (TAAA) [77]. Their retrospective study comprised 34 patients who received fenestrated endovascular stent graft repair (fEVAR) for thoracoabdominal aortic disease (19 had aortic dissection and 15 had thoracic abdominal aortic aneurysms). CT angiographic images were used to create patient-specific models which were printed using materials for biocompatibility and transparency. The aortic stent graft was deployed in each 3D printed model in the operating room to determine the exact position of each fenestration or branch which was marked on the stent graft (Figure 14 and Figure 15). This allows for accurate planning of personalised fEVAR based on measurements of the size of the fenestrations for individual arterial branches, thus reducing the risk of complications such as endoleak. Following customization, the fenestrated stent graft was reloaded into the delivery sheath for patient development. Results showed the successful treatment of these complicated thoracoabdominal aortic disease by customized fenestrated stent grafts with a mean follow-up of 8.5 months in this case series. All of the bridged visceral vessels were patent with no enlargement of the aneurysm during the follow-up [77]. This study proves the clinical value of 3D printed aortic models in guiding treatment of aortic disease by fEVAR, although further research on a large sample size with long follow-up of clinical outcomes is warranted.

### 3.5. 3D Printing in Pulmonary Artery Disease

Application of 3D printing in pulmonary diseases mainly lies in the pre-surgical planning for treatment of congenital heart disease involving anomalies such as pulmonary atresia or stenosis or coronary-pulmonary artery fistula [78,79,80,81]. With patient-specific 3D printed models incorporated into the management plan, it was found useful to assist decision making and reduce the contrast volume during angiography and improve the performance efficiency by interventional cardiologists.

An emerging area is to use 3D printing for detection of pulmonary embolism aiming to develop optimal CT pulmonary angiography (CTPA) protocols [82,83,84]. Our experience has shown its value in this aspect. A 3D printed pulmonary artery model using normal CTPA images was generated comprising the main pulmonary arteries [82]. The model was scanned on a 64-slice CT scanner with different protocols. There was high accuracy between the 3D printed model and original CT images with respect to replication of normal anatomy with mean difference between original CT images, STL and 3D printed model less than 5%. Low-dose CTPA protocol such as use of 80 kilovoltage peak (kVp) with 0.9 pitch was recommended with resultant lower radiation dose while still acquiring diagnostic images. This preliminary finding was supported by later experiments with simulation of pulmonary embolism in the 3D printed model.

**Figure 16:**
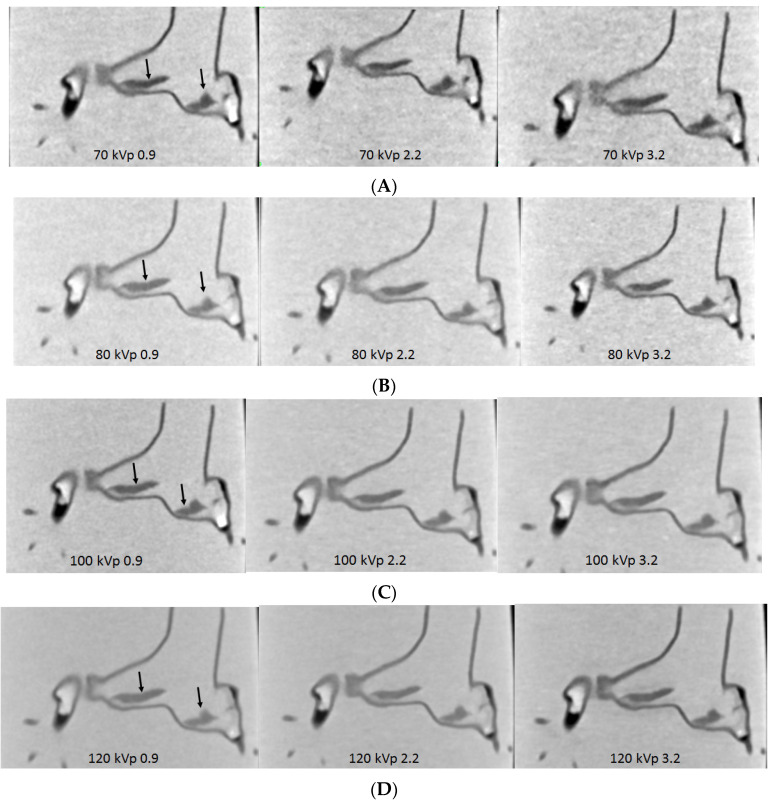
Computed tomography pulmonary angiography (CTPA) in the detection of main pulmonary embolism with different scanning protocols. (**A**,**B**) When pitch was increased to 3.2, image noise was increased with 70 and 80 kVp protocols; (**C**,**D**) in contrast, no significant change of image quality was noted with 100 and 120 kVp protocols, regardless of pitch values. Arrows refer to the thrombus in the main pulmonary arteries. Reprinted with permission under open access from Aldosari et al. [83].

**Figure 17:**
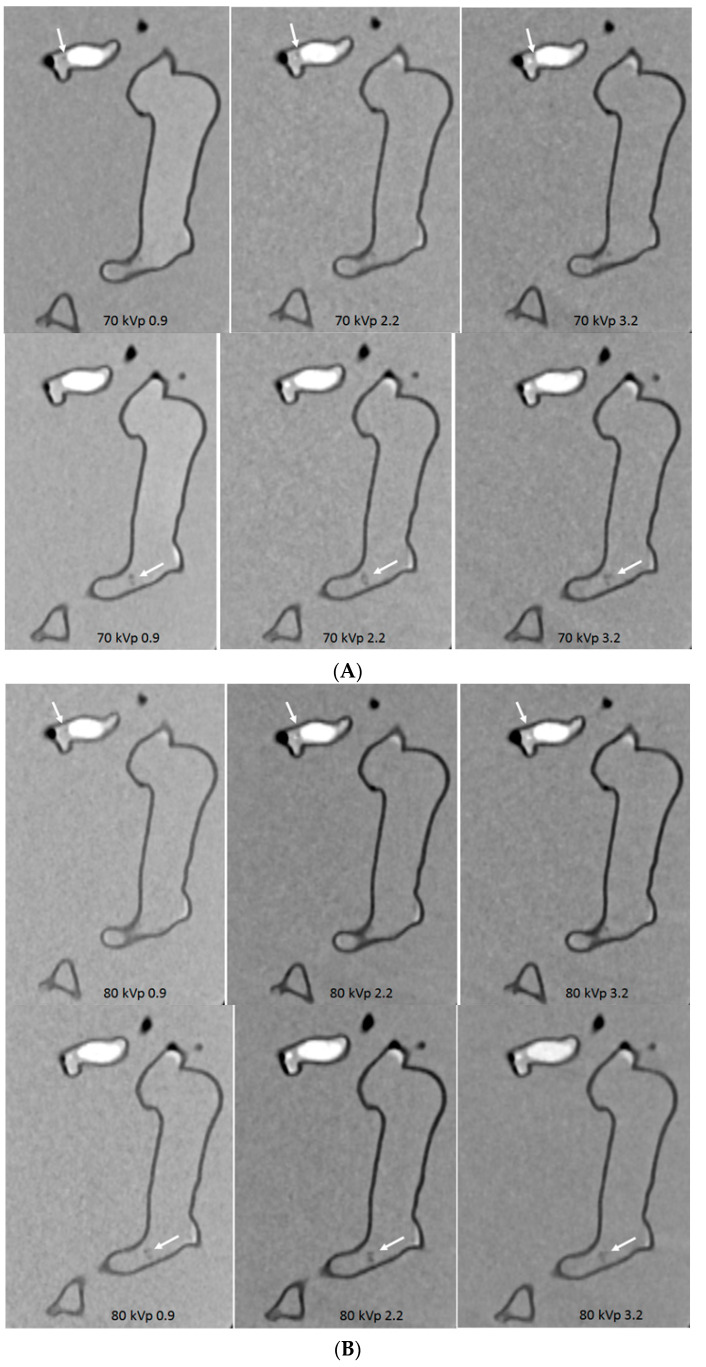

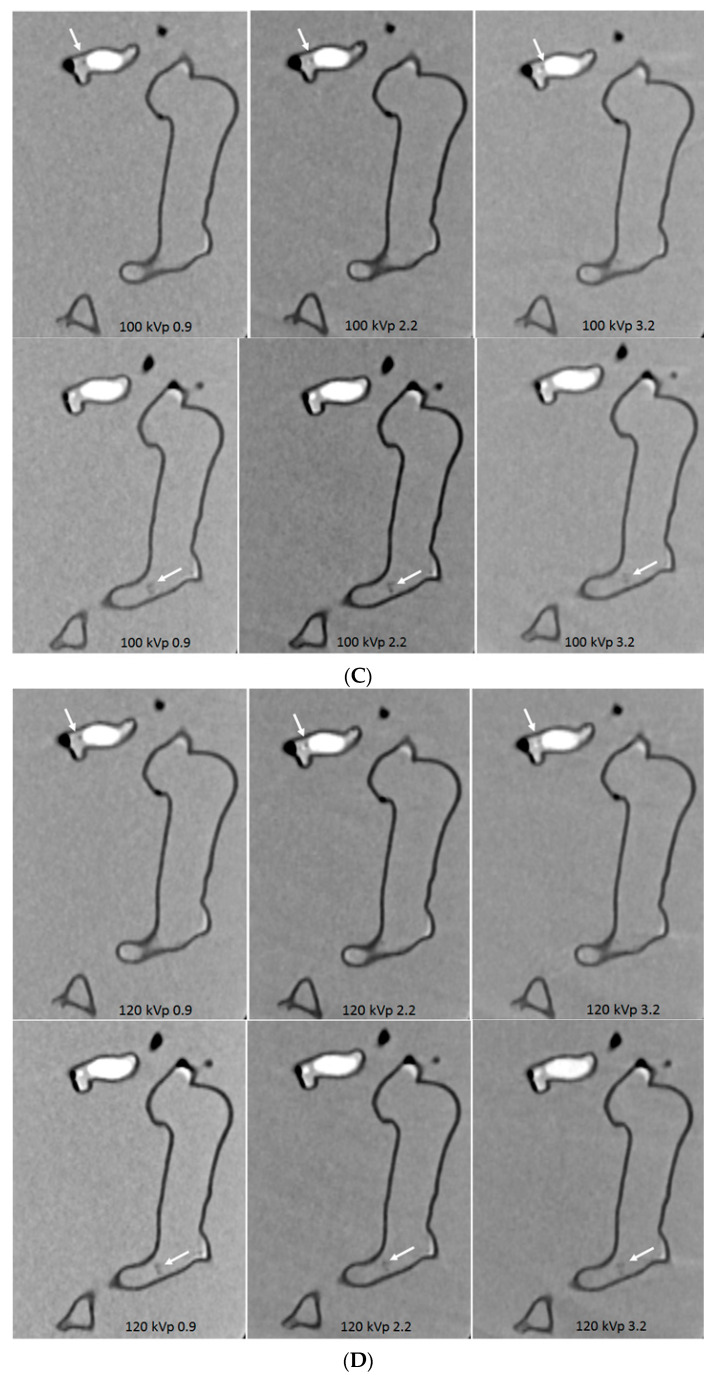
Computed tomography pulmonary angiography (CTPA) in the detection of peripheral pulmonary embolism with different scanning protocols. (**A**–**D**) The small thrombus is viewed as low-attenuation filling defect in the left segmental pulmonary artery (top arrows) and right pulmonary artery (bottom arrows) and they are visible in all protocols, regardless of kVp or pitch values used. Reprinted with permission under open access from Aldosari et al. [84].

With successful creation of personalized pulmonary artery model, we further tested different CTPA protocols using the 2nd and 3rd generation dual-source CT scanners. Large and small emboli were inserted into the main and side pulmonary arteries to mimic pulmonary embolism [83,84]. A series of CT scans were performed with kVp ranging from 70 to 80, 100 and 120, pitch 0.9, 2.2 and 3.2. Image quality was quantitatively analysed by measuring the signal-to-noise ratio and qualitatively assessed by two experienced radiologists. Results showed the feasibility of developing low-dose CTPA protocols when kVp was reduced from 120 to 100 or 80 and pitch was increased to 2.2 or 3.2 without significantly affecting the image quality when 128-slice CT was used (Figure 16) [83]. Whereas when CTPA was conducted on the latest CT such as 192-slice scanner, ultra low-dose protocol was also achievable when further reducing kVp to 70 and increasing pitch to 2.2 or 3.2 still allowing for detection of small and peripheral pulmonary embolism with no significant effect on the image quality (Figure 17) [84].

These findings indicate the potential applications of 3D printed models in optimizing CT scanning protocols, in particular in the diagnostic detection of pulmonary embolism, given the fact that CTPA is the first line technique in the diagnosis of pulmonary embolism. 3D printed realistic models offer advantages over commercial body phantoms not only because of low cost, but also the capability of providing individualized anatomy and pathology associated with 3D printed models as opposed to the commercial ones with only showing average adult or paediatric anatomical structures. Further research should focus on developing 3D printed chest phantom with simulation of bones, lungs, heart and cardiovascular vessels as well as muscles to represent realistic anatomical environment, thus protocols including chest and cardiac CT imaging could be optimized.

Another novel application of 3D printing technique is to assist management with the recent coronavirus disease 2019 (COVID-19) pandemic which results in a shortage of medical supplies, in particular, the personal protective equipment (PPE) [85,86,87,88], given the primary presentation of respiratory symptoms by COVID-19 [89,90,91,92,93], although it is also associated with cardiovascular disease [94,95,96,97]. 3D printing is playing an important role in providing medical devices including PPE when we are still fighting against the COVID-19 as shown in some reports documenting an overview of 3D printing applications and challenges in the COVID-19 [87,98].

### 3.6. 3D Bioprinting in Cardiovascular Disease

Bioprinting is promising, although it is still in its early stage. Tissue engineering combined with 3D printing represents a fast developing technique in recent years with creation of scaffold structures for use in regeneration of tissues and organs [99,100,101,102,103,104,105,106]. In recent years, significant progress has been made in 3D bioprinting with studies reporting promising results of printing cardiovascular tissues and organs.

Biomaterials guarantee the success of 3D bioprinting as materials must be biocompatible and non-toxic. Further, mechanical properties are equally important as they influence cell function and viability and cardiovascular structural integrity [107,108,109]. Bioinks used for bioprinting of cardiovascular tissues also need to mimic properties such as stiffness of the extracellular matrix of heart and other tissues [110].

Progress in 3D bioprinting and tissue engineering has made possible to generate 3D printed cardiovascular constructs, however, to achieve physiological levels of cardiomyocytes is still challenging with many obstacles to be overcome before printing tissues with the clinical sizes. Cell-laden bioprinting represents a very promising technology since it is capable of achieving well-controlled distribution of the cells (both cell density and diffusion distance) in a complex environment, mimicking the function of cardiovascular tissue and cardiomyocytes. Several studies have reported the feasibility of 3D bioprinting cardiovascular constructs, although the research was based on a small scale of vascular channels [111,112,113,114,115,116]. Miller et al. 3D printed a 1 mm diameter of vascular construct with generation of endothelial cell-line lumen but lack of perfusion to the constructs, thus, having very limited applications [111]. Kolesky and colleagues advanced the application to perfused channels in a 3D printed construct with human mesenchymal stem cells and fibroblasts embedded in an extracellular matrix [112]. They were able to inject the endothelial cells into the 3D printed channels and maintained perfusion for more than six weeks. Two research groups by Skylar-Scott and Brandenberg et al. used high-resolution laser methods to generate microvascularized constructs with a diameter of 20–50 μm simulating capillaries [113,114]. However, there is a strong demand on the use of multiplexing printheads since it requires very high resolution and fast printing to print this kind of scale scaffold. Jang et al. demonstrated the promising results of 3D printed complex vascular constructs with functional structures [115]. The 3D printed patch had 8 mm and 0.5 mm for diameter and height, respectively. In vivo animal testing, the developed stem cells improved cardiac functions, reduced cardiac fibrosis and scar formation, thus showing benefits in cardiac repair. Redd et al. presented the similar findings of developing 3D printed perfused microvascular constructs with implanted in the infarcted rat models [116]. The perfusable microvascular grafts as analysed by optical microangiography imaging showed improved vascular remodelling and enhanced coronary perfusion. These results reveal the promise and opportunity for cardiac tissue engineering combined with 3D bioprinting.

**Figure 18:**
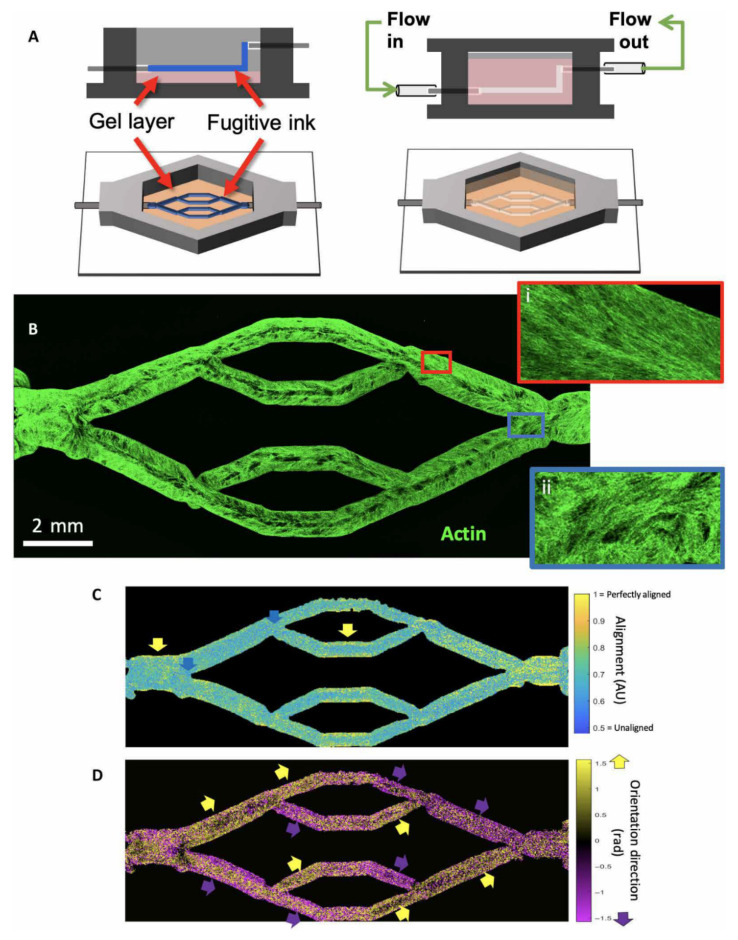
Bioprinting endothelialized vascular beds with complex geometry. (**A**) Schematic representation of vascular bed printing using the ink method. (**B**) Cytoskeletal morphology of microvascular endothelial cells shows high alignment in straight regions of the vascular bed (inset i, red border), with chaotic cytoskeletal alignment in the fork regions (inset ii, blue border). (**C**) Alignment of cytoskeleton in perfused engineered beds shows high actin alignment in the wall of straight vascular regions (yellow arrows), whereas fork regions exhibit weak alignment (blue arrows). AU, arbitrary units. (**D**) Direction of preferential alignment follows flow direction. Coloured arrows indicate principle flow direction (up, yellow; down, purple), which tends to agree with orientation direction of endothelial cell alignment. Reprinted with permission under open access from Hynes et al. [117].

Hynes et al. further extended the application of bioprinting to understand the dynamics of circulating tumour cells (CTC) within the vascular beds [117]. They used 3D bioprinting technology to develop living models of the human vasculature channels that are responsive to mechanical stress enabling analysis of biological and physical factors in vitro situation. The biomaterial was planted with human brain microvasculature endothelial cells and were then perfused to generate vascular geometry structures simulating normal tissues with cell growth and formation. CTC cell lines of a metastatic mammary gland carcinoma were chosen to ensure metastatic vascular attachment. Computational fluid modelling was performed to analyse hemodynamic changes such as wall shear stress. Figure 18 shows the steps of 3D printed vascular beds with complex geometry that were embedded in a hydrogel. Results showed the significant mechanical changes in relation to vessel distensibility with presence of endothelial cells. Particle image velocity (PIV) was used to analyse flow behaviour difference between acellular and endothelialized vessels in comparison with computational simulations. There was a good agreement between predicted and experimentally determined flow patterns for these two vessels. PIV observed the flow partitioning of CTC cells toward the centre of the vessel. This study offers a unique experimental design using a combination of different novel approaches including 3D bioprinting, bioengineering and computation for studying the biophysics of endothelial cell behaviour of CTC during metastasis.

## 4. Limitations and Future Research Directions

Promising results of 3D printing in the cardiovascular applications are shown in the above-mentioned areas, however, a number of limitations exist which still hinder the widespread use of 3D printing in clinical practice. Prospective and multi-centre studies are still lacking with RCTs only available in the 3D printing in CHD for medical education, while in other areas, case reports or case series dominate most of the applications (66%) as shown in a recent review article on 3D printing in CHD [118]. Inclusion of a larger sample size with potential longitudinal studies of mid to long-term outcomes is necessary to validate the clinical value of 3D printing such as how 3D printed models change patient’s management and whether treatment outcomes are significantly improved with incorporation of 3D printing into practice. High cost is another issue that needs to be addressed before 3D printing is incorporated into routine clinical practice. With cost reductions in 3D printers and printing materials, 3D printing will become an acceptable technique for both patients and clinicians.

Despite high accuracy and quality of 3D printed models, even with multicolour materials, most of the current models are static, thus they do not represent realistic properties similar to normal tissues. Generation of realistic and dynamic models is of clinically important for cardiovascular disease as simulation of hemodynamic blood flow in these 3D cardiovascular models plays a crucial role in creating a realistic environment that can mimic the real physiological situation. Some recent studies already demonstrated the dynamic mechanical properties of 3D printing materials in coronary artery disease and coronary stents [119,120,121]. 3D printed bioresorbable stents using polymer materials such as polylactic acid (PLA) or polycaprolactone (PCL) have shown good compatibility with blood and cells, and they can be used to print coronary stents for treating coronary artery disease [120]. Guerra et al. produced 3D printed composite stents using PLA and PCL materials. They showed that both PLA and PCL are both biocompatible with PLA stent presenting with an average cell proliferation of 8.28% and PCL 12.46% after three days [121]. However, each of them has limitations with PLA having high modulus (high elasticity with excellent recoil ratio) but low properties restricting its implantation, while PCL having low modulus (high rigidity with best radial expansion), thus limiting its application for stenting purpose. Therefore, composite PCL/PLA stents were recommended as they improve the performance by overcoming each material’s limitations.

Although extensive attempts have been made by researchers to use 3D bioprinting technologies to print heart, blood vessels, heart valves as well as cardiovascular constructs [111,112,113,114,115,116,122,123,124,125,126], the main technological barriers or obstacles in bioprinting are to develop suitable biomaterials (bioinks) with good mechanical strength and biocompatibility to match the biological properties of native organs [127]. Bioprinting of cardiovascular organs or cells is more challenging since the bioprinted cells/stem cells need to maintain proliferation and differentiation in a living extracellular microenvironment. Despite good resolution available with the current bioprinters, further improvement in resolution is necessary to print small vessels with high accuracy. Table 1 is a summary of bioprinting processes showing comparison of different types of bioprinters, printing materials and other features. These promising findings will need to be tested in vivo situations before bioprinted models can be applied to patients for treating cardiovascular disease.

## 5. Summary

There is no doubt that 3D printing represents a fast evolving technology over the last decades with increasing reports available in different medical domains. Use of 3D printing in cardiovascular disease is one of the most exciting applications in the medical field, given the high prevalence of cardiovascular disease worldwide and associated high morbidity and mortality. Personalized 3D printed models revolutionize the current practice of planning and treating patients with different kinds of cardiovascular disease, ranging from congenital heart disease to acquired coronary, valvular, aortic and pulmonary artery diseases. These realistic physical models enhance clinician’s understanding of complex pathological situations in relation to the surrounding structures and improve their confidence in performing difficult and complex procedures, hence achieving the outcomes of better patient management with low surgery or procedure-related complications. 3D bioprinting is showing its significant impact on the development of 3D printing technology with early results of printing biocompatible cardiovascular tissues and structures. This article provides an overview of the clinical applications of 3D printing in these areas. With further improvements in 3D printing technology and advancements in printing materials in the near future, we expect to see more promising results available in the literature and witness how this technique benefits patient care and clinical practice.

## Figures and Tables

**Table 1:** Characteristics of 3D bioprinting processes. Reprinted with permission under open access from Li et al. [127].

Types of Bioprinters	Biomaterials	Cell Viability/Resolution	Bioprinting Speed	Cost	Advantages	Disadvantages
Inkjet-based bioprinting	Low-viscosity suspension of living cells; biomolecules; growth factors	~90% 20–100 μm	Fast (<10,000 droplets/s)	Low	Wide availability; low cost; high resolution; high printing speed; ability to introduce concentration gradients in 3D constructs	Poor vertical structure clogging characteristics; thermal and mechanical stress to cells; limited printable materials (liquid only)
Pressure-assisted bioprinting	Hydrogel; melt; cells; proteins and ceramic materials; solutions, pastes, or dispersions of low to high viscosity; Poly Lactic-co-Glycolic Acid (PLGA); tricalcium phosphate (TCP); collagen and chitosan; collagenalginate-silica composites coated with HA; and agarose with gelatin	40–80% 200 μm	Slow	Medium	Numerous materials that can be printed with any dimensions; mild conditions (room temperature); use of cellular spheroids; direct incorporation of cells; and homogenous distribution of cells	Limited mechanical stiffness; critical timing of gelation time; specific matching of the densities of the material and the liquid medium to preserve shapes; low resolution and viability
Laser-assisted bioprinting	Hydrogel, media, cells, proteins and ceramic materials of varying viscosity	>95% >20 μm	Medium	High	Nozzle-free, noncontact process; cells are printed with high activity and high resolution; high control of ink droplets and precise delivery	High cost; cumbersome and time consuming; requires a metal film and thus is subject to metallic particle contamination
Stereolithography	Light-sensitive polymer materials; curable acrylics and epoxies	>90% ~1.2–200 μm	Fast (<40,000 mm/s)	Low	Solid freeform and nozzle-free technology; highest fabrication accuracy; compatibility with an increasing number of materials; light-sensitive hydrogels can be printed layer-by-layer	Applicable to photopolymers only; lack of biocompatible and biodegradable polymers; harmful effects from residual toxic photo-curing reagents; possibility of harm to DNA and human skin by ultraviolet (UV)
